# Supplementary material for: High-resolution crystal structure of a 20 kDa superfluorinated gold nanocluster
Source: Nat Commun. 2022 May 11;13:2607. doi: 10.1038/s41467-022-29966-2 (PMC9095690; doi:10.1038/s41467-022-29966-2)
Supplement: Supplementary file 1 — Supplementary Information File [file 41467_2022_29966_MOESM1_ESM.pdf]

Supplementary Information

High-Resolution Crystal Structure of a 20 kDa Superfluorinated Gold Nanocluster

Claudia Pigliacelli,[1,2] Angela Acocella,[3] Isabel Díez,[2] Luca Moretti,[4] Valentina Dichiarante,[1]* Nicola Demitri,[5] Hua Jiang,[2] Margherita Maiuri,[4] Robin H. A. Ras,[2,6] Francesca Baldelli Bombelli,[1] Giulio Cerullo,[4] Francesco Zerbetto,[3] Pierangelo Metrangolo,[1,2]* Giancarlo Terraneo[1]*

[1] *Laboratory of Supramolecular and Bio-Nanomaterials (SupraBioNanoLab), Department of Chemistry, Materials, and Chemical Engineering "Giulio Natta", Politecnico di Milano, via L. Mancinelli 7, 20131 Milano, Italy.*

[2] *Department of Applied Physics, Aalto University School of Science, Puumiehenkuja 2, FI-00076 Espoo, Finland.*

[3] *Dipartimento di Chimica "G. Ciamician", Università di Bologna, V. F. Selmi 2, 40126, Bologna, Italy.*

[4] *IFN-CNR, Dipartimento di Fisica, Politecnico di Milano, 20133 Milano, Italy.*

[5] *Elettra-Sincrotrone Trieste S.S. 14 Km 163.5 in Area Science Park, 34149 Basovizza, Trieste, Italy.*

[6] *Department of Bioproducts and Biosystems, Aalto University School of Chemical Engineering, P.O. Box 16000, FI-00076 Espoo, Finland.*

* E-mail: valentina.dichiarante@polimi.it; pierangelo.metrangolo@polimi.it; giancarlo.terraneo@polimi.it.

Supplementary Method 1

Starting materials were purchased from Sigma-Aldrich, Fluorochem, Apollo Scientific and used as received. Commercial HPLC-grade solvents were used without further purification. Solkane® (1,1,1,3,3- pentafluorobutane) was purchased from Solvay and filtered through a 0.2 μm PTFE membrane before use. Milli-Q water was obtained by a Simplicity (Millipore) instrument.

Analytical thin layer chromatography (TLC) was performed on precoated silica gel 60 F254 plates and visualization was done by staining with 0.2 M aqueous $KMnO_4$.

DSC analyses were performed on a Mettler Toledo DSC823e differential scanning calorimeter, using aluminum 40 μL sample pans and Mettler STARe software for calculation. Melting points were also determined on a Reichert instrument, by observing the melting process through an Olympus BH-2 optical microscope.

^{1}H- and ^{19}F-NMR spectra were recorded on a Bruker AV400 spectrometer operating at 400 MHz for both nuclei. A coaxial capillary tube containing deuterated benzene (C_6D_6) was used for the lock.

Supplementary Method 2

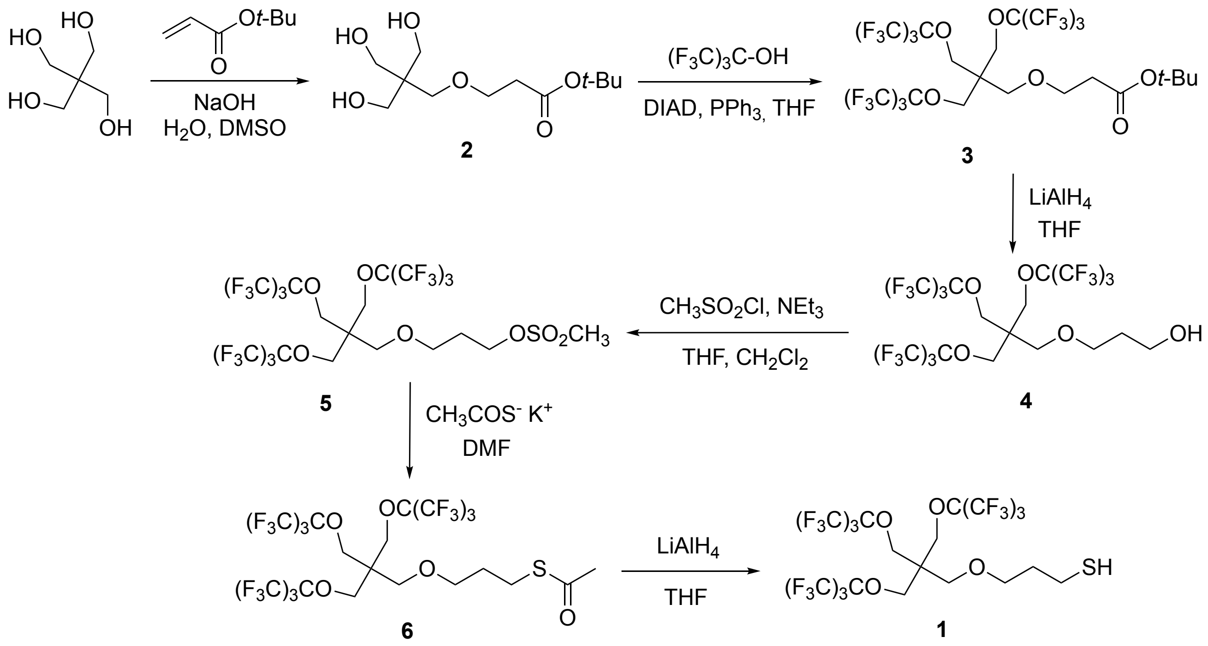

Supplementary Figure 1. Scheme of Thiol $F_{27}SH$ synthesis procedure.

***tert*-Butyl 3-(3-hydroxy-2,2-bis(hydroxymethyl)propoxy)-propanoate (2)**

Following the same procedure described by Yue,[S1] compound **2** was obtained in 38% yield as a colorless oil, eluting with CH_2Cl_2/CH_3OH (from 98:2 to 9:1).

^{1}H NMR (500 MHz, $CDCl_3$) δ: 3.69 (t, 2H, *J* = 5.8 Hz), 3.67 (s, 6H), 3.56 (s, 2H), 2.48 (t, 2H, *J* = 5.8 Hz), 2.35 (brs, 3H), 1.46 (s, 9H).

***tert*-Butyl 3-(3-((1,1,1,3,3,3-hexafluoro-2-(trifluoromethyl)-propan-2-yl)oxy)-2,2-bis(((1,1,1,3,3,3- hexa-fluoro-2-(trifluoromethyl)propan-2-yl)oxy)methyl)propoxy)propanoate (3)**

Triphenylphosphine (30 g, 115 mmol) and 4 Å molecular sieves (3.5 g) were placed in an oven-dried 500 mL flask, under nitrogen flow. Compound **2** (6 g, 23 mmol) and anhydrous THF (150 mL) were added and the solution was cooled to 0 °C. Diisopropylazodicarboxylate (DIAD, 22 mL, 115 mmol) was added dropwise under stirring. The resulting foamy mixture was warmed up to r.t. and stirred for additional 20 minutes, before adding perfluoro-*tert*-butyl alcohol (16 mL, 115 mmol) in one portion. The finally clear solution was stirred at 45 °C for 65 hours. After cooling down to r.t. and removing the molecular sieves by filtration, water (100 mL) was added and stirring was continued for 10 more minutes. Dichloromethane (50 mL) was added and the lower organic phase was separated, dried over Na_2SO_4 and concentrated under vacuum. Flash silica gel chromatography of the crude with hexane/ethyl acetate (98:2) gave product **3** as a colorless solid (9.5 g, 45% yield).

m.p. 76-78 °C. **^{1}H NMR** (500 MHz, $CDCl_3$) δ: 4.06 (s, 6H), 3.64 (t, 2H, *J* = 6.5 Hz), 3.43 (s, 2H), 2.45 (t, 2H, *J* = 6.5 Hz), 1.44 (s, 9H). **^{19}F NMR** (475 MHz, $CDCl_3$) δ: -71.41 (s).

3-(3-((1,1,1,3,3,3-Hexafluoro-2-(trifluoromethyl)propan-2-yl)oxy)-2,2-bis(((1,1,1,3,3,3-hexafluoro- 2-(trifluoromethyl)-propan-2-yl)oxy)methyl)propoxy)propan-1-ol (4)

Compound **3** (3 g, 3.3 mmol) was treated with $LiAlH_4$ (360 mg, 9.5 mmol) in anhydrous THF (45 mL) according to the reported procedure.[S1] Purification of the crude by SiO_2

chromatography with hexane/ethyl acetate (8:2) afforded alcohol **4** as a colorless oil (2.3 g, 83% yield).

^{1}H NMR (500 MHz, $CDCl_3$) δ: 4.05 (s, 6H), 3.72 (t, 2H, *J* = 6.2 Hz), 3.54 (t, 2H, *J* = 6.2 Hz), 3.40 (s, 2H), 1.82 (qui, 2H, *J* = 6.2 Hz). **^{19}F NMR** (475 MHz, $CDCl_3$) δ: -71.41 (s).

3-(3-((1,1,1,3,3,3-Hexafluoro-2-(trifluoromethyl)propan-2-yl)oxy)-2,2-bis(((1,1,1,3,3,3-hexafluoro- 2-(trifluoromethyl)-propan-2-yl)oxy)methyl)propoxy)propyl methanesulfonate (5)

Reaction of **4** (3 g, 3.5 mmol) with triethylamine (1.4 mL, 10 mmol) and methanesulfonyl chloride (0.8 mL, 10 mmol) in a mixture of anhydrous THF (15 mL) and anhydrous DCM (30 mL) was performed as described by Yue.[S1] Flash chromatography over silica gel with hexane/ethyl acetate (8:2) as eluent yielded **5** as a colorless oil (3.1 g, 97% yield).

^{1}H NMR (500 MHz, $CDCl_3$) δ: 4.27 (t, 2H, *J* = 6.3 Hz), 4.06 (s, 6H), 3.53 (t, 2H, *J* = 6.1 Hz), 3.42 (s, 2H), 2.98 (s, 3H), 2.00 (qui, 2H, *J* = 6.3 Hz). **^{19}F NMR** (475 MHz, $CDCl_3$) δ: -71.45 (s).

(*S*)-(3-(3-((1,1,1,3,3,3-Hexafluoro-2-(trifluoromethyl)propan-2-yl)oxy)-2,2bis (((1,1,1,3,3,3-hexafluoro-2-(trifluoromethyl)-propan-2yl)oxy)methyl)propoxy)propyl) ethanethioate (6)

A solution of **5** (2.8 g, 3 mmol) in DMF (28 mL) was treated with potassium thioacetate (1.1 g, 9 mmol) at 50 °C, as reported.[S1] Silica gel chromatography, eluting with hexane/ethyl acetate (95:5), gave product **6** as a pale yellow oil (2.3 g, 85% yield).

^{1}H NMR (500 MHz, $CDCl_3$) δ: 4.06 (s, 6H), 3.43 (t, 2H, *J* = 6.1 Hz), 3.38 (s, 2H), 2.89 (t, 2H, *J* = 7.1 Hz), 2.32 (s, 3H), 1.83 (qui, 2H, *J* = 6.6 Hz). **^{19}F NMR** (475 MHz, $CDCl_3$) δ: -71.48 (s).

3-(3-((1,1,1,3,3,3-Hexafluoro-2-(trifluoromethyl)propan-2-yl)oxy)-2,2-bis(((1,1,1,3,3,3-hexafluoro- 2-(trifluoromethyl)-propan-2-yl)oxy)methyl)propoxy)propane-1-thiol (1)

Compound **6** (2 g, 2.2 mmol) was treated with $LiAlH_4$ (230 mg, 6 mmol) in anhydrous THF (25 mL), according to Yue's procedure.[S1] Flash chromatography with hexane/ethyl acetate (98:2) afforded thiol **1** as a colorless solid (1.5 g, 81% yield).

m.p. 39-40 °C. ^{1}**H NMR** (400 MHz, $CDCl_3$) δ: 4.06 (s, 6H), 3.50 (t, 2H, *J* = 6.1 Hz), 3.40 (s, 2H), 2.56 (dd, 2H, *J* = 7.2 and 15 Hz), 1.89-1.82 (m, 2H), 1.32 (t, 1H, *J* = 7.9 Hz). ^{13}**C NMR** (100 MHz, $CDCl_3$) δ: 120.2 (q, *J* = 293 Hz), 79.7 (m), 69.7, 66.2, 65.7, 46.2, 33.6, 21.2. ^{19}**F NMR** (475 MHz, $CDCl_3$) δ: - 71.57 (s). **FTIR** (cm^{-1}): 1490, 1468, 1243, 1189, 1153, 1122, 1011, 969, 913, 771, 736, 725, 538, 515, 478. **MS (ESI+)** *m/z*: 887 [M+Na]. **HRMS for** $\mathbf{C_{20}H_{15}F_{27}O_4S}$: calcd. 864.025994; found 864.023560.

Supplementary Method 3

To provide a comprehensive description of the interactions involved in the **$[Au_{25}(SF_{27})_{18}]^0$** crystal structure, we use quantum mechanical calculations to investigate the stabilization energy and the bonding nature of fluorine-fluorine interactions, with the aim to unravel their role in the supramolecular assembly.

In particular, in order to gain an estimate of the intermolecular energy in agreement with previous literature data, we performed scans of intermolecular F-F separations on a CF_4 stable dimer, chosen as smaller reference model of a two-points C-F----F-C interaction, here defined as VV0 following the Mahlanen's nomenclature,[S2] showing a halogen-halogen intermolecular dihedral angle of 0° in D3h symmetry.

Intermolecular energy scans were carried on with the Minnesota 2006 hybrid meta exchange-correlation functional (M06-2X),[S3] suitable to reproduce intermolecular Van der Waals interactions, in combination with aug-cc-pvtz basis set[S4,S5] on fluorine atoms and TZVP basis set[S6] on carbons. Grimme's GD3 Empirical dispersion calculations[S7] were used, also corrected with the basis set superposition error (BSSE)[S8] by means of Gaussian16 Suite of programs.[S9]

Moreover, intra- and inter-Wiberg bond orders[S10] BO_{AB}, between two fluorine atoms A and B, were calculated from the off-diagonal elements of the electron density matrix, $P_{\mu\upsilon}$, calculated from the Lowdin orthonormalized M06-2x wavefunctions of the CF_4 complex, according to the following equation:

$$BO_{AB} = \sum_{\mu\in A,\nu\in B} P_{\mu\upsilon}P_{\upsilon\mu} \qquad 1.$$

The M06-2x functional provides an estimate of the interaction energy in the VV0 dimer of about - 0.280 kcal/mol, in very good agreement with previous reported values, calculated at MP2/aug(df)-6-311G* level of theory[S11] and similar to other weak noncovalent interactions. The calculated intra (between fluorine atoms of the same CF_4) and inter F-F bond orders (between fluorine atoms belonging to two nearby CF_4) are 0.32 and 0.032 arb. units, respectively. The latter is relatively small, if compared to covalent or hydrogen bonds, indicating a small electron delocalization between fluorine atoms.[S12]

The calculated intermolecular potential energy surface on the VV0 dimer was then fitted by means of a modified Morse potential (Eq. 2), in the form successfully applied by Mahlanen *et al.* to map intermolecular interaction in tetrahalide dimers.

$$E(r)=-\boldsymbol{\varepsilon}(1-(1-e^{-A(r-r^*)})^2 \qquad 2.$$

The resulting fitted parameters, i.e., $\boldsymbol{\varepsilon}$=0.274 (curve depth), A=1.645 (slope of repulsion) and r*=3.085 (the equilibrium distance between faced fluorine atoms) are in very good agreement with the Mahlalen's data. Supplementary Figure S3 shows the fitted energy curve.

In order to unambiguously quantify the intermolecular energy stabilization of C-F···F-C interactions involved in and between nearby **F_{27}S-** ligand molecules when arranged in crystal structures, the parametrized intermolecular potential was then applied to cartesian coordinates of $C(CF_3)_3$ groups extracted from perfecta-thiols branches of **F_{27}SH** and **$[Au_{25}(SF_{27})_{18}]^0$**, and saturated with H atoms (see Supplementary Figure S3). In particular, by distinguishing F···F interactions as: i) intra CF_3, ii) intra-branches, iii) inter-branches, iv) inter-perfecta and v) inter-cluster in the Au-NPs structure (see Figures 1a and 4a of the main text), our analysis provides a clear and complete mapping of non-covalent interactions in the investigated crystal structures. As Figure 1b and 4a of the main text show, despite the intra-CF_3 interaction energy (red points) results destabilizing, probably due to steric hindrance, all other F···F interactions locally impart a not-negligible stabilization to both investigated crystal structures. In **$[Au_{25}(SF_{27})_{18}]^0$** we can observe an increasing of destabilizing F···F contacts in both intra-CF_3 (red points) and inter-branches interactions (green points), but however largely overwhelmed by a cumulative stabilization due to the other F···F interactions. It is worth to note how, in the Au-NPs, the inter-cluster fluorine-fluorine interactions result stabilizing at all occurring distances.

Moreover, the calculation of inter- and intra- bond orders between all F···F couples allows us to classify their interaction according to the degree of the electron density delocalization occurring between perfecta arms. As Supplementary Figure 4 shows, F···F bond order decreases as the interatomic distances increase (Supplementary Fig. 4a and b) while, its correlation with the intermolecular energy reveals the typical behaviour of "*close-contacts*" interactions, as also reported for other fluorinated compounds,[S13] *i.e.*, an excessive accumulation of the electron density between two fluorine atoms (higher than 0.08 arb. units) destabilizes the interaction, unlike a typical strong shared interaction such as the C-C bond.[S14]

The above presented F···F bond order analysis was also corroborated by a non-covalent interaction (NCI) study. In particular, by applying the NCI 4 plotting program[S15,S16] on the M06-2X wavefunctions calculated on the perfecta-thiol branches extracted from **F_{27}SH** and **$[Au_{25}(SF_{27})_{18}]^0$** crystal structures, we provide an additional, clear visualization and classification of non-covalent weak interactions as attractive and repulsive.

The NCI approach bases on the evaluation of the non-covalent interaction index correlated to second eigenvalue (λ_2) of the electron-density Hessian (second derivative) matrix, whose sign is directly related to the interaction type, i.e., for bonding interactions, $\lambda_2 < 0$, for non-bonded interactions, such as steric repulsion, $\lambda_2 > 0$, while for van der Waals interactions, characterized by a negligible density overlap, $\lambda_2 \lesssim 0$. The 2d maps presented in the main text in Figures 1d (for two nearby thiol-molecules in F_{27}SH structure), and 4d and 4e (for two nearby clusters in **$[Au_{25}(SF_{27})_{18}]^0$**) show plots of the reduced electron density gradient versus the electron density multiplied by the sign of λ_2, sign(λ_2)ρ, providing a direct mapping of different types of weak interactions present in the investigated structures. The conventional color code applied uses blue to indicate attractive (negative values of sign(λ_2)ρ) interactions, red for repulsive (positive values of sign(λ_2)ρ) interactions and green for weak Van-der Waals interactions. The corresponding gradient isosurfaces shown in Figures 1e and f of the main manuscript are colored accordingly.

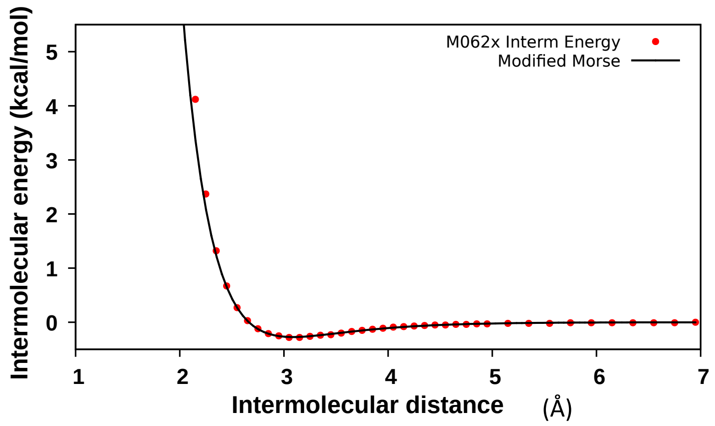

Supplementary Figure 2. Fitted potential energy surface of VV0 dimer calculated at M06-2x level of theory, with aug-cc-pvtz basis set on fluorine atoms and TZVP basis set on carbons, in combination with Grimme's GD3 Empirical dispersion calculations and basis set superposition error (BSSE) correction.

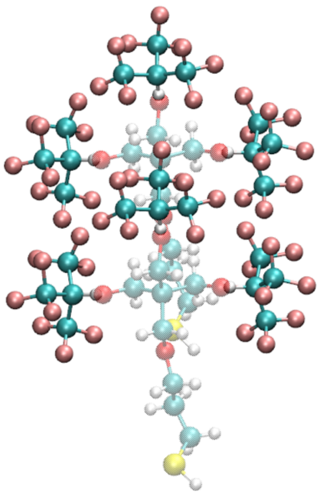

Supplementary Figure 3. 3D representation of hydrogen-saturated $C(CF_3)_3$ groups of super-fluorinated branches in two nearby fluorinated-thiols as extracted from the $\mathbf{F_{27}SH}$ crystal coordinates. To assist the eye, the complete fluorinated-thiols are also shown, displayed by a transparent representation.

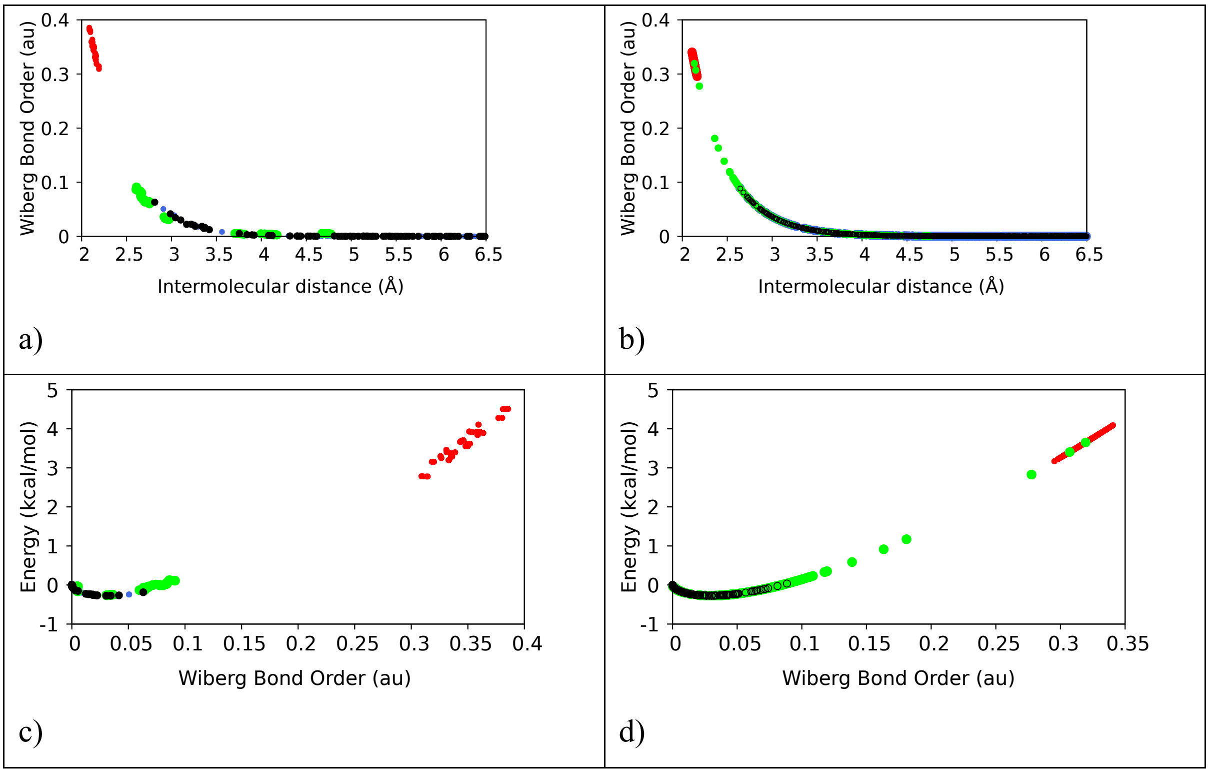

Supplementary Figure 4. Correlation between Wiberg bond order calculated at M062x/aug-cc-pvtz level of theory with F---F internuclear distance in a) $\mathbf{F_{27}SH}$ and b) $\mathbf{[Au_{25}(SF_{27})_{18}]^0}$ and with F···F intermolecular energy in c) $\mathbf{F_{27}SH}$ and d) $\mathbf{[Au_{25}(SF_{27})_{18}]^0}$. Color code: red intra CF_3, green F···F *intra*-branches, blue *inter*-branches, and black for F-F *inter*-perfecta

The main electronic structures presented in the manuscript were calculated on the Cartesian coordinates of $C(CF_3)_3$ groups extracted from perfecta-thiols branches of $F_{27}SH$ and $[Au_{25}(SF_{27})_{18}]$ in their respective crystallographic structures and saturated with H atoms. No optimizations of the crystal structure were performed; no molecular dynamics were run.

Relaxed potential energy surface scans on the intermolecular F ••• F distance of the two facing fluorine atoms were carried on the CF_4 dimer, with steps of ± 0.1 Å each. Here, we report the initial Cartesian coordinates of the stable dimer, optimized at PBE0/aug-cc-pvtz level of theory[S17,S18] by means of Gaussian16 suite of programs[S19], used to perform the energy surface scan:

C	-2.89428400	0.00142200	0.00027900
F	-3.35179700	-1.07400700	0.60759600
F	-3.28562300	-0.00481700	-1.25728800
F	-1.57846300	0.00791200	0.05063800
F	-3.36207300	1.07662400	0.60013300
C	2.89427000	-0.00143300	-0.00025600
F	1.57844500	-0.00812400	0.04988500
F	3.28576800	0.00608400	-1.25776900
F	3.36219200	-1.07712600	0.59860200
F	3.35156200	1.07346500	0.60817700

Supplementary Method 4

Fluorinated gold nanoclusters were obtained by a modified Brust reaction, as previously reported by Dass and coworkers.[S20] Briefly, in a round-bottomed flask, tetraoctylammonium bromide (82 mg, 0.15 mmol, 1 eq) was dissolved in α,α,α-trifluorotoluene (30 mL). $HAuCl_4 \cdot 3H_2O$ (60 mg, 0.15 mmol, 1 eq) was separately dissolved in the minimum amount of Milli-Q water and added under vigorous stirring to the organic solution, which turned orange upon addition. The selected thiol was dissolved in 1 mL of α,α,α-trifluorotoluene and added to the reaction mixture, which was then stirred for additional 10 minutes, until colorless, and

then cooled to 0 °C with an ice bath. An ice-cold solution of $NaBH_4$ (57 mg, 1.5 mmol, 10 eq) in Milli- Q water (4 mL) was added dropwise and the resulting mixture was vigorously stirred at 0 °C for 3 hours. The obtained dark brown organic phase was washed with Milli-Q water (3×25 mL) and evaporated at reduced pressure, at room temperature. The residue was then taken up with the minimum amount of toluene and transferred into a Falcon tube for purification. Purification was performed by repeating the following steps:

- ultrasound treatment (59 kHz) for 10 minutes
- centrifugation for 10 minutes (8694 rcf for **1**; 1717 rcf for PFDT)
- removal of the supernatant
- addition of clean toluene

After the last purification cycle, the supernatant was analyzed by ^{19}F and ^{1}H NMR (20% v/v $CDCl_3$ added for lock) to check the absence of excess of reagents. The remaining pellet was dried and dissolved in the minimum amount of solkane®. The resulting dark solution was sonicated for 10 minutes, filtered through a 0.2 µm PTFE filter and stored at room temperature in screw-capped glass vials.

Supplementary Method 5

Dark prismatic crystals were grown by slow evaporation of a diluted solkane solution (two months, room temperature). Spontaneous crystallization afforded $[Au_{25}(SF_{27})_{18}]^0$ clusters in amounts ranging from 25 to 30 % yield (based on starting Au).

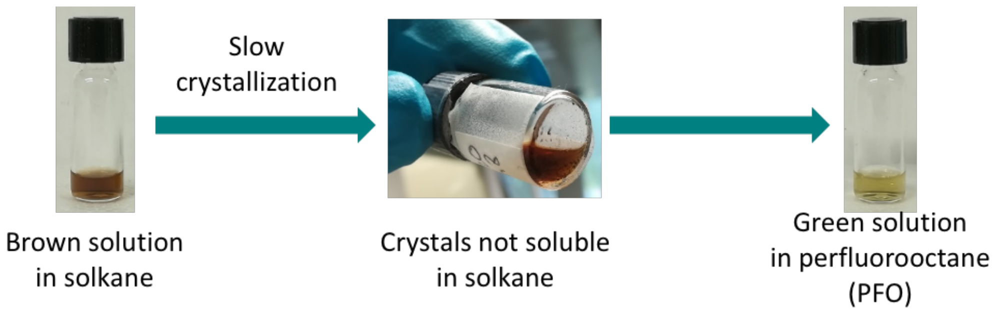

Supplementary Figure 5: Images of crude product in solkane, crystal formation and crystal solution in PFO.

Supplementary Method 6

UV-vis spectra of fluorinated gold nanoclusters bulk solution in solkane and of $\mathbf{[Au_{25}[(SF_{27})_{18}]^0}$ crystals redissolved in PFO at the appropriate dilution were acquired at room temperature on a Agilent Cary 5000 UV-Vis spectrometer.

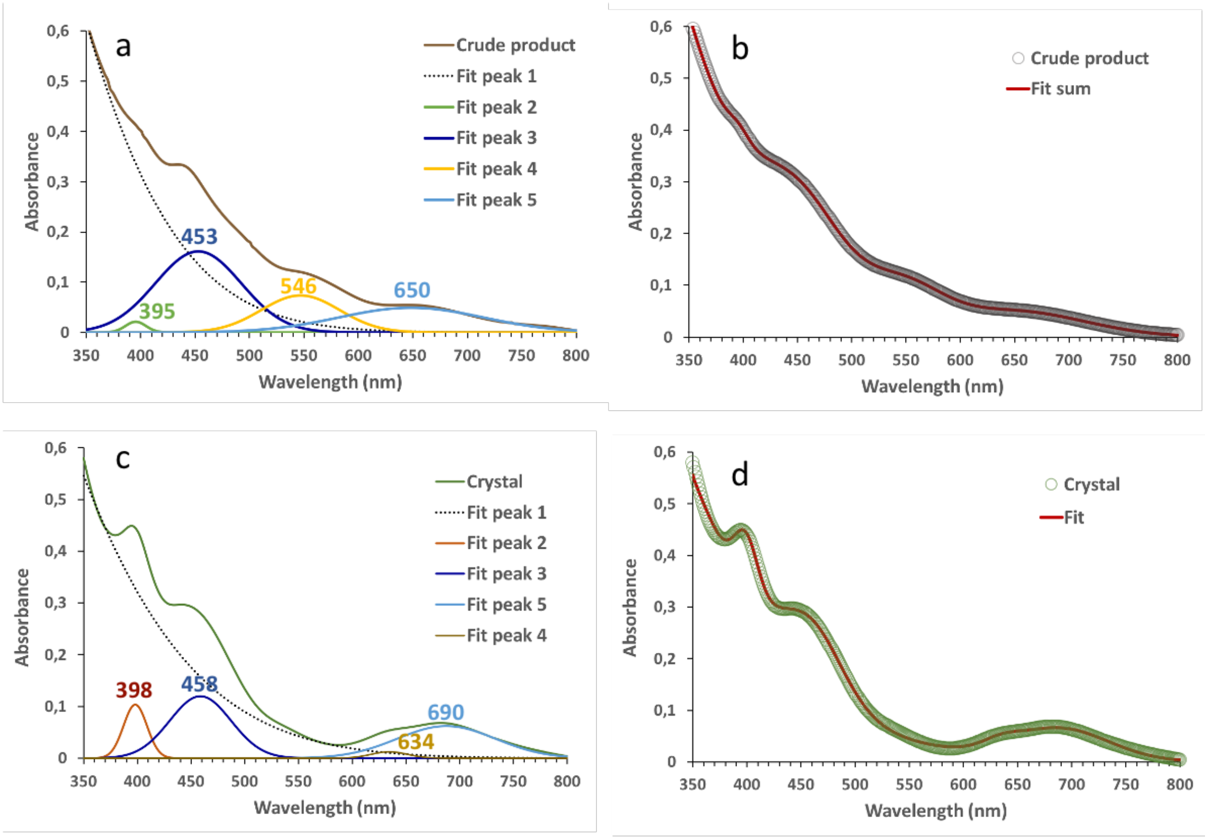

Supplementary Figure 6: (a) UV-vis spectrum (brown line) of crude product in solkane. The absorption spectrum is fitted to a sum of Gaussians (showed below the spectrum). The fifth Gaussian (dotted line) is to take the general UV rise into account. The peak maximum is denoted above each Gaussian. (b) Sum of Gaussian fitting for UV-Vis spectrum of the crude product. (c) UV-vis spectrum (green line) of crystal in PFO. The absorption spectrum is fitted to a sum of Gaussians (showed below the spectrum). The fifth Gaussian (dot line) is to take the general UV rise into account. The peak maximum is denoted above each Gaussian. (d) Sum of Gaussian fitting for UV-Vis spectrum of the crystal sample dissolved in PFO.

Supplementary Method 7

High-resolution scanning transmission electron microscopy (STEM) measurements were carried out with a JEOL 2200FS double aberration corrected FEG TEM/STEM, operating at 200 kV. Solutions were dropped onto a TEM grid (carbon films 400 mesh Au).

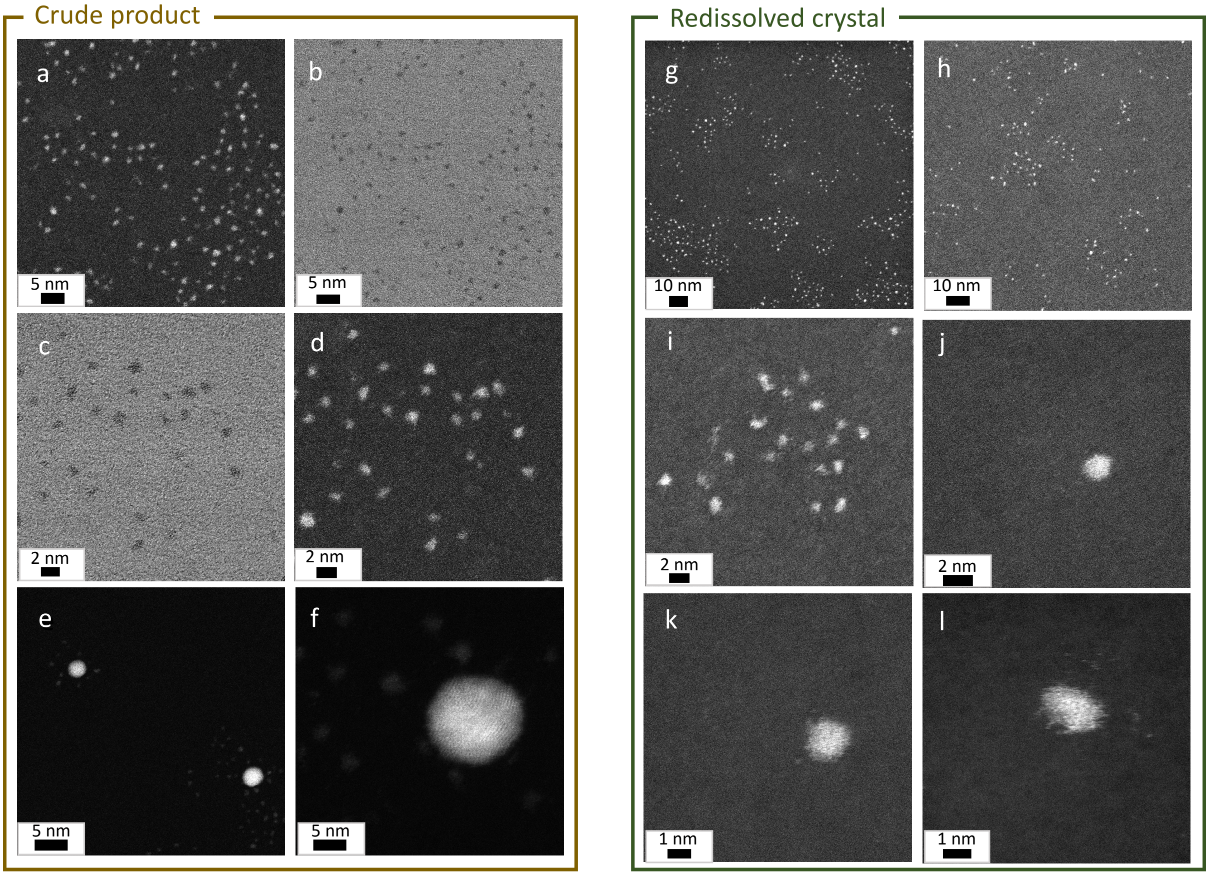

Supplementary Figure 7: STEM images of crude product (a-f) and redissolved crystal (g-l).

Supplementary Method 8

An Autoflex II instrument from Bruker Daltoniks (Bremen, Germany) equipped with a UV/N2-laser (337 nm/100 lJ) was used to carry out MALDI analyses. 2,3,4,5,6-Pentafluorobenzoic acid dissolved in solkane was used as the matrix. The crystallized fluorinated nanoclusters and the matrix, both dissolved in solkane, were mixed in a 1:1 (v/v) ratio and applied on the stainless steel target plate in 1 μL aliquots. The sample spot was dried in air at room temperature. The mass spectrum (4-20 kDa) was measured in linear positive-ion mode, typically performing 1500 scans, and Protein standard solution II (Bruker Daltonics) was used for the external molecular mass calibration.

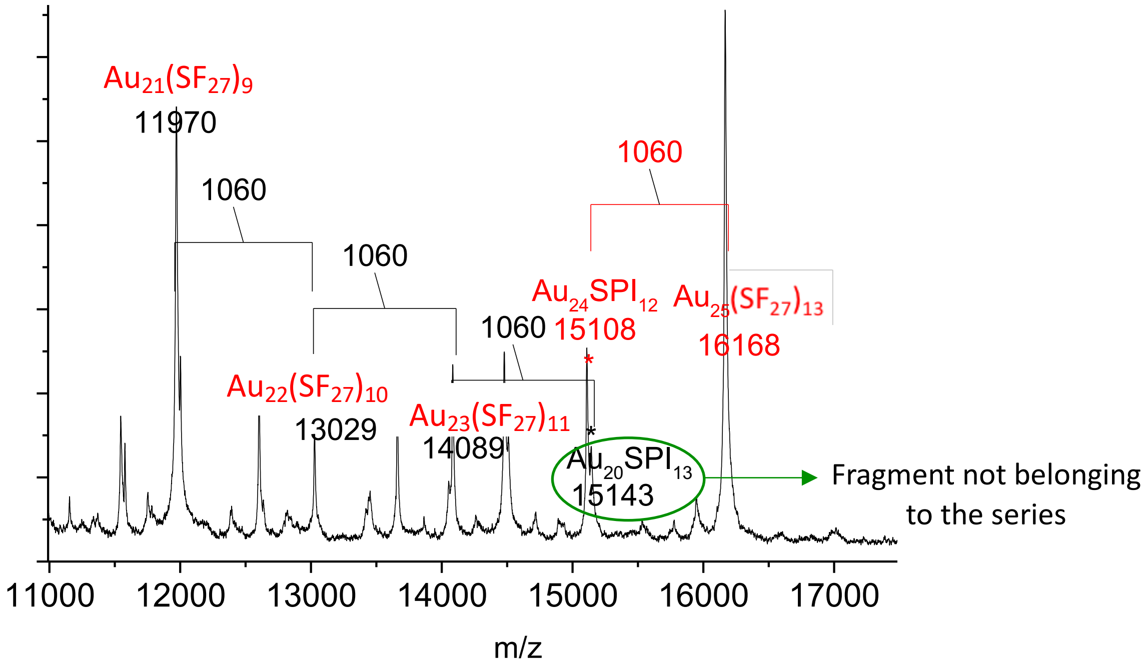

Supplementary Figure 8: MALDI spectrum of **$Au_{25}(SF_{27})_{18}$** (crystal). The expected parent peak (20464.9 m/z) could not be observed. A first fragment is observed at 16168 m/z, followed by a fragmentation pattern indicating the gradual loss of 1 atom of Au and 1 thiol molecule (1060 m/z) under the employed laser pulse. One additional fragment, not belonging to the series, could also be observed at 15143 m/z.

Supplementary Method 9

NMR analysis has been performed on crystal samples that were previously tested on the single crystal X-ray diffractometer. All tested crystals showed the same cell parameters.

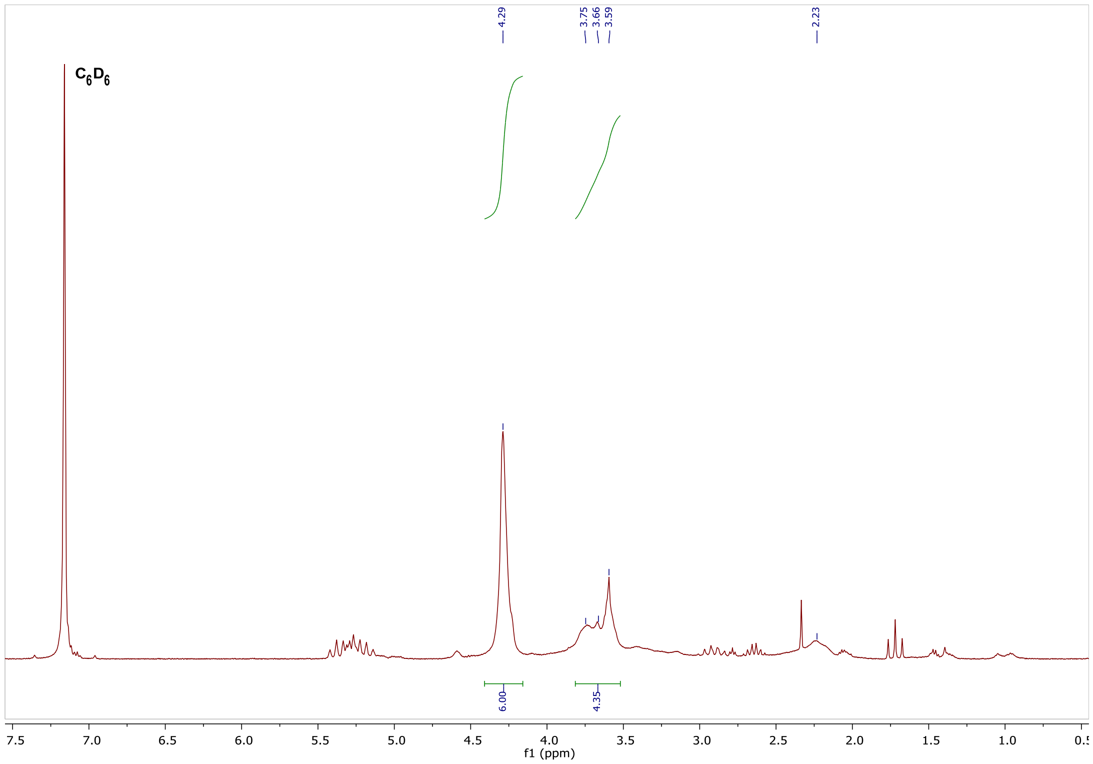

Supplementary Figure 9: ^{1}H-NMR spectrum of clusters dissolved in PFO (400 MHz; lock: C_6D_6). Signals in the regions 6-5 ppm and 1.5-0 ppm are due to trace impurities of PFO solvent. [For comparison ^{1}H-NMR spectrum of $F_{27}SH$ dissolved in PFO is listed herein (δ, ppm): 4.29 (s, 6H); 3.63 (t, 2H); 3.59 (s, 2H); 2.62 (q, 2H); 1.92 (t, 2H); 1.12 (t, 1H)].

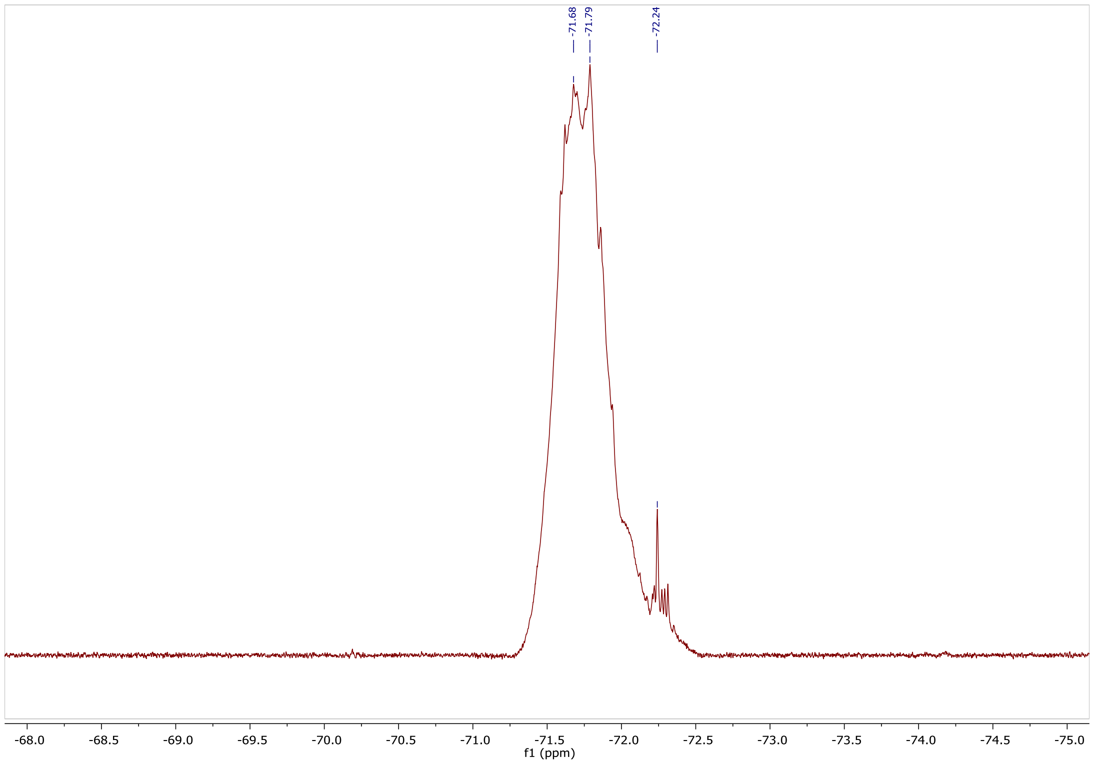

Supplementary Figure 10: ^{19}F-NMR spectrum of clusters dissolved in PFO (400 MHz; lock: C_6D_6; scan window: 10 ppm; offset: -72 ppm). [For comparison, ^{19}F-NMR spectrum of PFO and $F_{27}SH$ dissolved in PFO are listed herein. PFO (δ, ppm): -83.5 (t, 3F, J = 10.3 Hz); -123.4 (bs, 2F); -124.3 (bs, 2F); -128.0 (bs, 2F). $F_{27}SH$ (δ, ppm): -72.3 (s)].

As reported (see ref. S28 and S30) gold atomically precise nanoclusters coated by fluorinated ligands could show multiple peaks in the ^{19}F NMR spectra due to the different chemical environment experienced by ligand molecules and thus by their CF_3 groups in according to their binding site on the metal core. Similarly, for our system a broad and complex multiplet is detected in the chemical shift range typical of $F_{27}SH$ ligand. Due to the different chemical environment experienced by CF_3 groups separation of single peaks was not possible and, in addition, the complexity of the signal is even higher probably due to the interference of the perfluorinated solvent, PFO.

Supplementary Method 10

Data collections were performed at the X-ray diffraction beamline (XRD1) of the Elettra Synchrotron, Trieste (Italy)[S21]. The crystals were dipped in NHV oil (Jena Bioscience, Jena, Germany) and mounted on the goniometer head with kapton loops (MiTeGen, Ithaca, USA). Complete datasets were collected at 100 K through the rotating crystal method. Data were acquired using a monochromatic wavelength of 0.700 Å, on a Pilatus 2M hybrid-pixel area detector (DECTRIS Ltd., Baden-Daettwil, Switzerland). The diffraction data were indexed and integrated using XDS.[S22] Semi-empirical absorption correction and scaling was performed, exploiting multiple measures of symmetry-related reflections, using SADABS program.[S23] The structures were solved by the dual space algorithm implemented in the SHELXT code.[S24] Fourier analysis and refinement were performed by the full-matrix least-squares methods based on F^2 implemented in SHELXL (Version 2017/1).[S28] The Coot program was used for modeling.[S29] Anisotropic thermal motion refinement have been used for all atoms with occupancies greater than 50%. Hydrogen atoms were included at calculated positions with isotropic $U_{factors} = 1.2 \cdot U_{eq}$ (U_{eq} being the equivalent isotropic thermal factor of the bonded non hydrogen atom). Restrains on bond lengths, angles and thermal motion parameters (DFIX, DANG and SIMU) have been applied on disordered ligand fragments. Electron content of cavities have been estimated with the SQUEEZE routine of PLATON.[S30] No ordered solvent molecules could be modeled in the asymmetric unit (ASU) of $\mathbf{Au_{25}(SF_{27})_{18}}$, therefore not

construable residual density has been squeezed (563 electrons in 8% - 1278 Å^3 - of the unit cell volume). The disordered solvent has been estimated as additional three solkane solvent molecules in the ASU (1,1,1,3,3-pentafluorobutane or 1,1,1,2,3,3,3-heptafluoropropane). Essential crystal and refinement data are reported below (Table 1S).

Supplementary Table 1. Crystallographic data and refinement details for **$Au_{25}(SF_{27})_{18}$**.

	**$Au_{25}(SF_{27})_{18}$** [$Au_{25}(C_{20}H_{14}F_{27}O_4S)_{18}$]
Chemical Formula	$C_{360}H_{252}Au_{25}F_{486}O_{72}S_{18}$
Formula weight (g/mol)	20464.84
Temperature (K)	100(2)
Wavelength (Å)	0.700
Crystal system	Triclinic
Space Group	*P* -1
Unit cell parameters	a = 26.683(5) Å<br>b = 26.879(5) Å<br>c = 28.337(6) Å<br>α = 117.14(3)°<br>β = 91.10(3)°<br>γ = 117.54(3)°
Volume (Å^3)	15338(7)
Z	1
Density (calculated) (g·cm^{-3})	2.216
Absorption coefficient (mm^{-1})	5.945
F(000)	9625
Crystal size (mm^3)	0.30 x 0.10 x 0.10
Crystal habit	Thick black prisms
Theta range for data collection	0.83° to 25.27°
Resolution (Å)	0.82
Index ranges	$-32 \leq h \leq 32$<br>$-32 \leq k \leq 32$<br>$-34 \leq l \leq 34$
Reflections collected	235248
Independent reflections (data with I>2σ(I))	57592 (47628)
Data multiplicity (max resltn)	4.04 (4.03)
I/σ(I) (max resltn)	11.04 (6.97)
R_{merge} (max resltn)	0.0692 (0.1647)
Data completeness (max resltn)	98.5% (99.0%)
Refinement method	Full-matrix least-squares on F^2
Data / restraints / parameters	57592 / 3478 / 5452
Goodness-of-fit on F^2	1.069
Δ/σ_{max}	0.084
Final R indices [I>2σ(I)]^a	R_1 = 0.0619,<br>wR_2 = 0.1780
R indices (all data)^a	R_1 = 0.0707,<br>wR_2 = 0.1862

Largest diff. peak and hole (e·Å^{-3})	3.413 and -2.566
R.M.S. deviation from mean (e·Å^{-3})	0.213
CCDC number	2045605

^{a} $R_1 = \Sigma ||Fo|–|Fc|| / \Sigma |Fo|$, $wR_2 = \{\Sigma [w(Fo^2 – Fc^2)^2] / \Sigma [w(Fo^2)^2]\}^{½}$

Supplementary Table 2. Interatomic distance in Au_{25} core

Atom	Atom	Length/Å
Au1_10	Au2_10[1]	2.7802(13)
Au1_10	Au2_10	2.7802(13)
Au1_10	Au4_10	2.795(2)
Au1_10	Au4_10[1]	2.795(2)
Au1_10	Au5_10[1]	2.7786(9)
Au1_10	Au5_10	2.7786(9)
Au1_10	Au7_10	2.7805(8)
Au1_10	Au7_10[1]	2.7806(8)
Au1_10	Au8_10[1]	2.7998(7)
Au1_10	Au8_10	2.7997(7)
Au1_10	Au9_10[1]	2.7851(10)
Au1_10	Au9_10	2.7851(10)
Au2_10	Au3_10	3.0263(9)
Au2_10	Au4_10[1]	3.0702(9)
Au2_10	Au5_10[1]	2.7871(16)
Au2_10	Au6_10	3.1608(18)
Au2_10	Au7_10	2.9766(9)
Au2_10	Au8_10	2.916(2)
Au2_10	Au9_10[1]	3.0308(8)
Au2_10	Au10_10[1]	3.2690(9)
Au3_10	Au5_10[1]	3.0587(10)
Au3_10	Au8_10	3.2273(11)
Au4_10	Au5_10[1]	2.8935(15)
Au4_10	Au6_10[1]	3.0494(11)
Au4_10	Au7_10[1]	2.7975(10)
Au4_10	Au8_10	2.8915(11)
Au4_10	Au9_10	2.9548(11)
Au4_10	Au11_10[1]	3.0509(10)
Au4_10	Au12_10	3.2277(9)
Au5_10	Au7_10	2.9096(10)
Au5_10	Au8_10[1]	2.9116(7)
Au5_10	Au9_10	3.0223(14)
Au5_10	Au10_10	3.0836(18)
Au5_10	Au11_10	3.188(2)

Atom	Atom	Length/Å
Au6_10	Au7_10	3.218(2)
Au7_10	Au8_10	3.0553(15)
Au7_10	Au9_10	2.962(2)
Au7_10	Au11_10	3.0977(13)
Au7_10	Au13_10	3.1569(12)
Au8_10	Au9_10	2.7861(10)
Au8_10	Au12_10	3.011(2)
Au8_10	Au13_10	3.0764(9)
Au9_10	Au10_10	3.1717(11)
Au9_10	Au12_10	3.0585(13)
Au9_10	Au13_10	3.2591(9)

[1]1-X,1-Y,1-Z

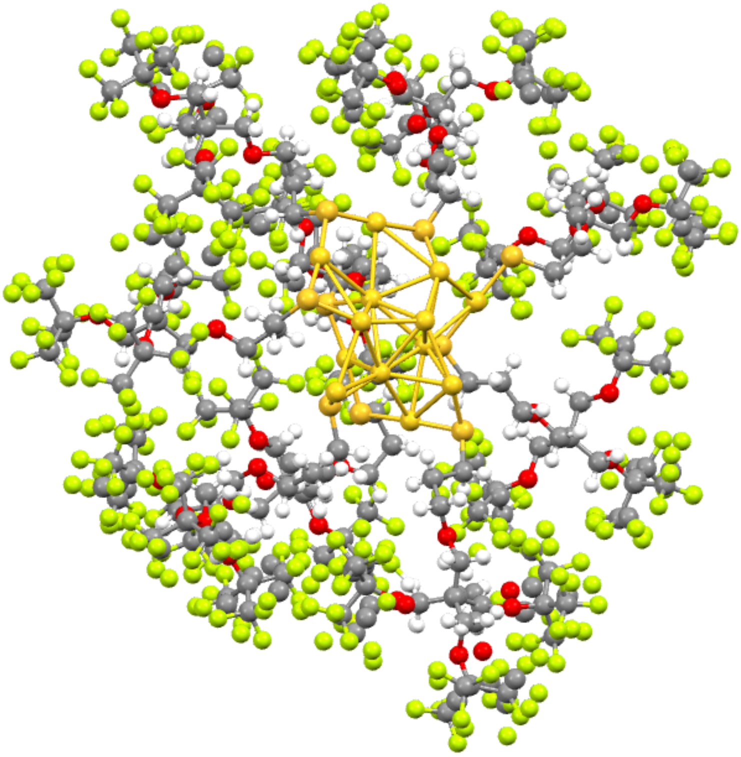

Supplementary Figure 11: Crystallographic representation (Mercury[S25] CSD 4.2.0, ball-and-stick model) of the asymmetric unit of **$Au_{25}(SF_{27})_{18}$**. Atom color code: Au: dark yellow, C: grey; O: red; S: yellow; F: pale yellow and H: white.

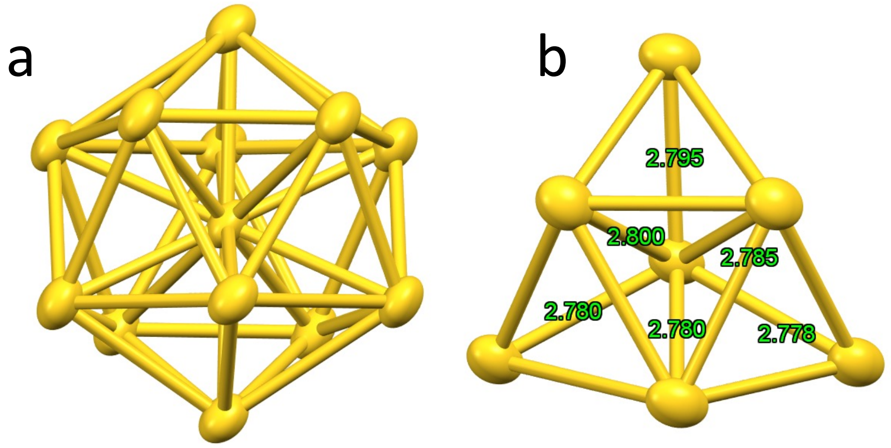

Supplementary Figure 12: Crystallographic representation (Mercury CSD 4.2.0, ellipsoid model) of: (a) the icosahedral core of **$Au_{25}(SF_{27})_{18}$**; (b) the asymmetric unit of the icosahedral core of **$Au_{25}(SF_{27})_{18}$** where the bond Au-Au distance (in Å) between the central Au atom and the six inner Au atoms are reported.

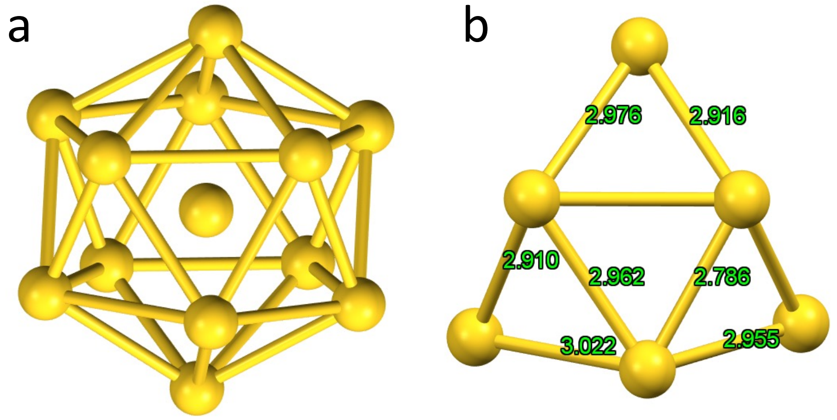

Supplementary Figure 13: Crystallographic representation (Mercury CSD 4.2.0, ball and stick model) of: (a) the icosahedral core of **$Au_{25}(SF_{27})_{18}$** where the 12 gold atoms are not bonded to the central Au atom; (b) the asymmetric unit of the icosahedral core of **$Au_{25}(SF_{27})_{18}$** without the central Au atom where the bond Au-Au distances (in Å) between the adjacent inner Au atoms are reported.

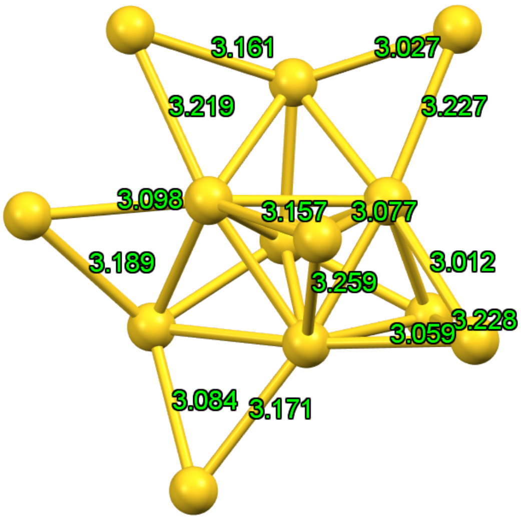

Supplementary Figure 14: Crystallographic representation (Mercury CSD 4.2.0, ball and stick model) of the asymmetric unit of the icosahedral core of $\mathbf{Au_{25}(SF_{27})_{18}}$ where the bond Au-Au distances (in Å) between the inner Au atoms and outer Au atoms are reported.

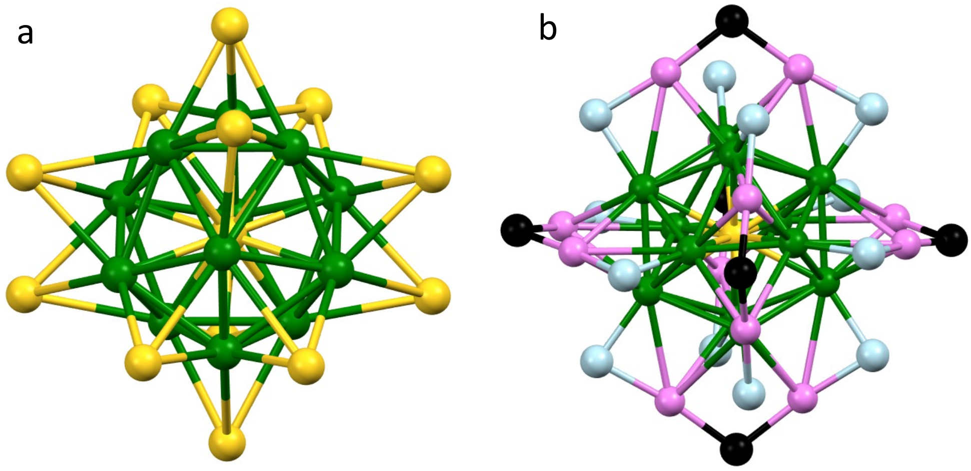

Supplementary Figure 15: Crystallographic representation (Mercury CSD 4.2.0, ball and stick model) of: (a) the star shape of the 25 gold core of $\mathbf{Au_{25}(SF_{27})_{18}}$ where the central inner atoms is colored in yellow the 12 Au atoms are colored in green and the 12 outer Au atoms are colored in yellow; (b) the fragment $\mathbf{Au_{25}S_{18}}$ where the color code for Au atoms is: Au_{cent} in yellow, Au_{inn} in green, Au_{out} in pink. The six S-Au_{out}-S-Au_{out}-S staple motifs are shown and the color code is for the sulphur atom is: S atom terminal in light blue and S atom bridged in black.

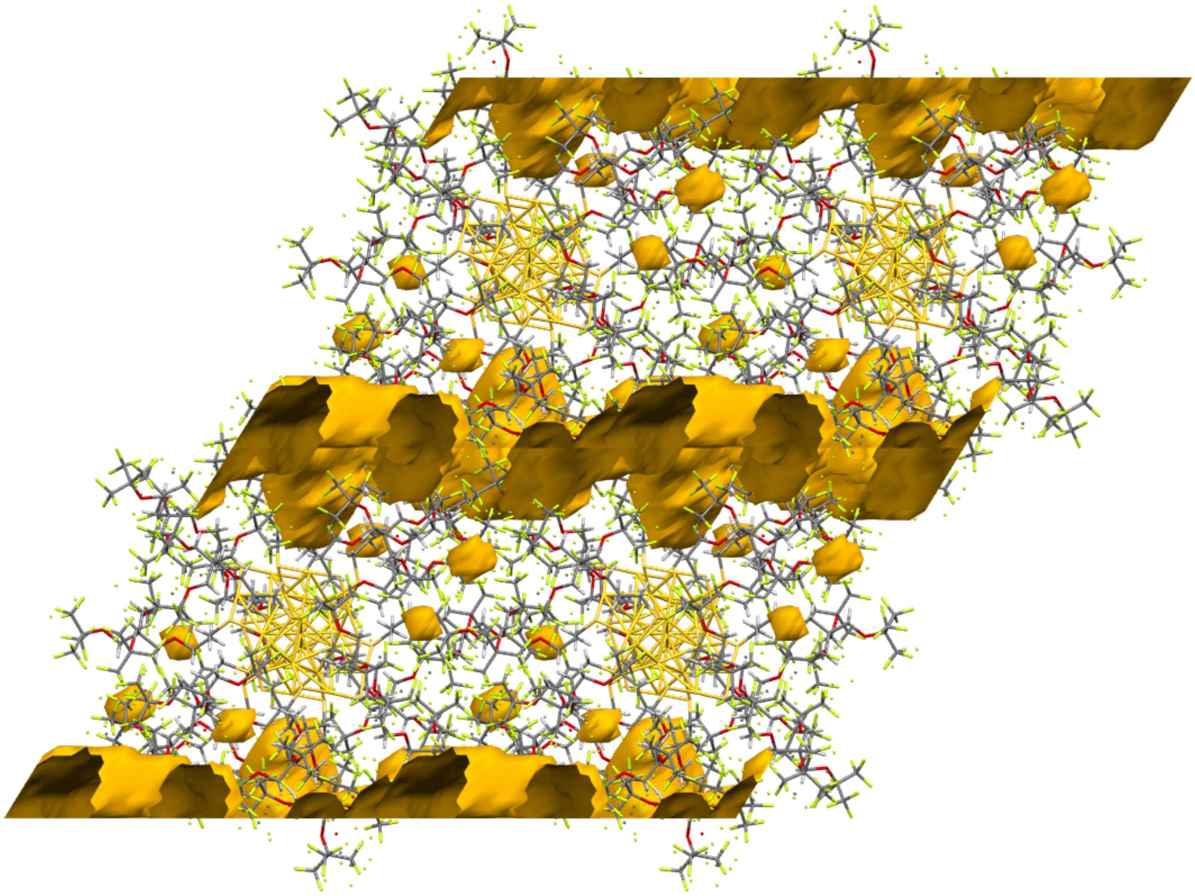

Supplementary Figure 16: Crystallographic representation (Mercury CSD 4.2.0, capped stick model) of **$Au_{25}(SF_{27})_{18}$** where the voids are shown. Electron content of cavities have been estimated with the SQUEEZE routine of PLATON. No ordered solvent molecules could be modeled in the asymmetric unit (ASU) of **$Au_{25}(SF_{27})_{18}$**, therefore not construable residual density have been squeezed (563 electrons in 8% - 1278 $Å^3$ - of the unit cell volume). The disordered solvent has been estimated as additional three solkane solvent molecules in the ASU (1,1,1,3,3-pentafluorobutane or 1,1,1,2,3,3,3-heptafluoropropane).

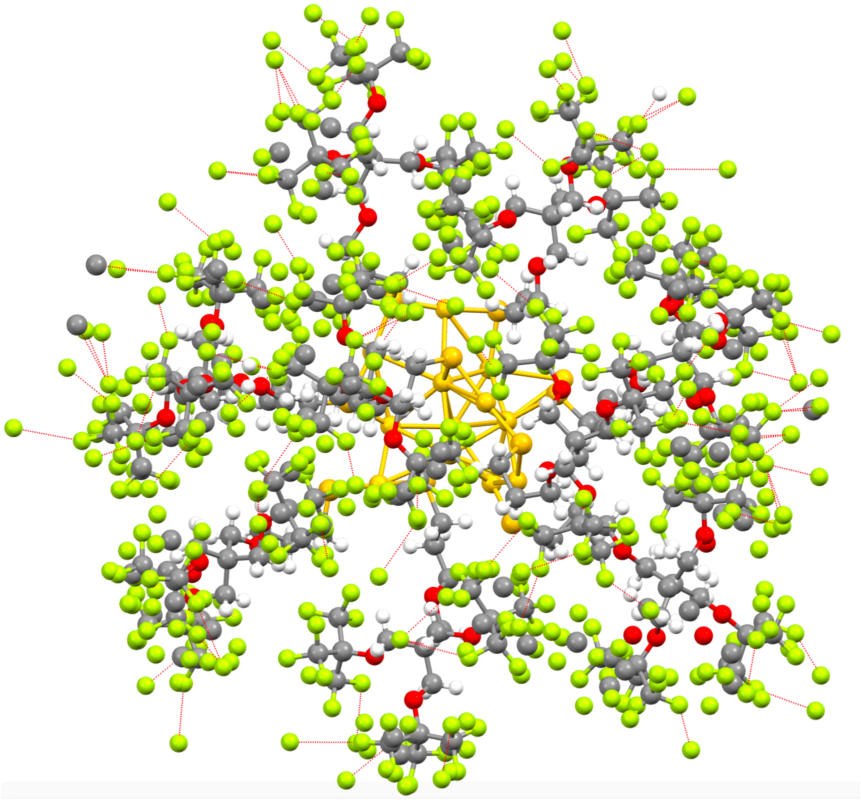

Supplementary Figure 17: Crystallographic representation (Mercury CSD 4.2.0, ball and stick model) of the asymmetric unit of **$Au_{25}(SF_{27})_{18}$** where the intermolecular contacts are shown as red dotted lines.

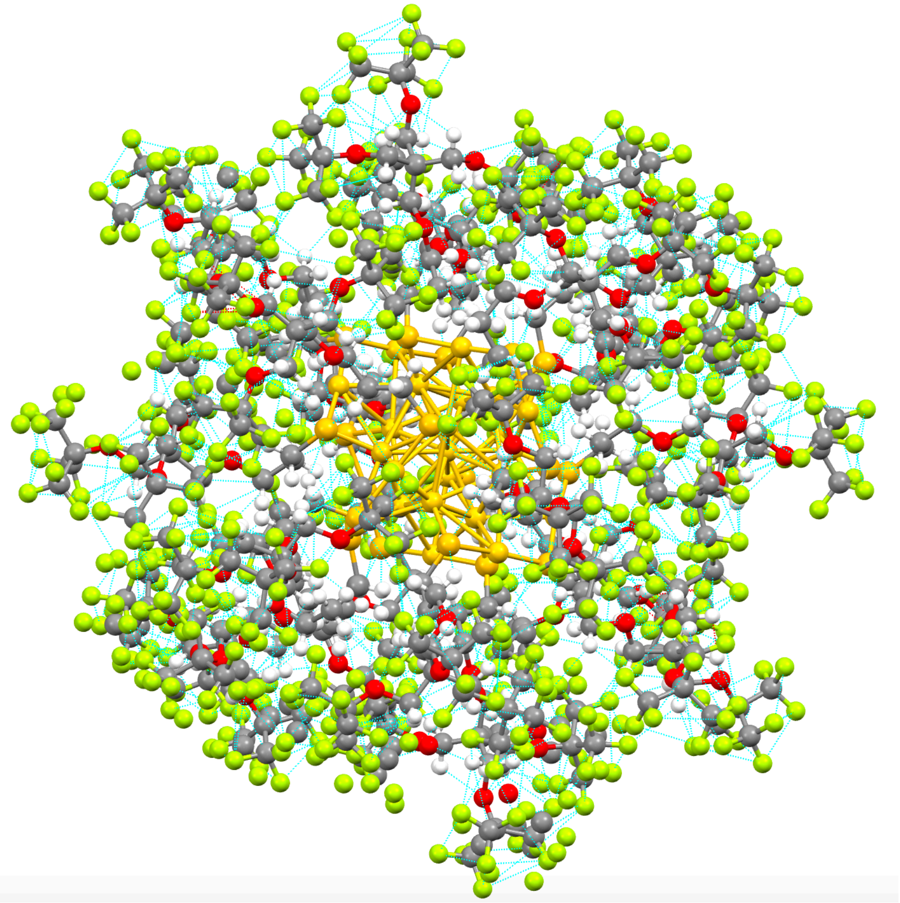

Supplementary Figure 18: Crystallographic representation (Mercury CSD 4.2.0, ball and stick model) of the of **$Au_{25}(SF_{27})_{18}$** where the intramolecular contacts (separated by > 3 bonds) are shown as light blue dotted lines.

Supplementary Method 11

Data mining in the Cambridge Structural Database[S26] (CSD 2021.01) was performed to reveal the solved crystal structures having a high number of fluorine atoms.

Search 1_ The query was built using Search by Formula applying the following parameters: $F_{>200}$ and Search on: each molecule in turn.

Output 1: 1 entry,

CCDC code: GUGSIN[S27], Formula $C_{510}H_{153}Ag_{112}Cl_6F_{306}{}^{3-}$, $3(C_7H_{17}ClN^+)$.

Search 2_ The query was built using Search by Formula applying the following parameters: $F_{>180.}$ Search on: each molecule in turn.

Output 2: 2 entries,

CCDC code: GUGSIN

CCDC code: UKOCAB[S31], Formula $C_{320}H_{96}Au_{67}Cl_4F_{192}{}^{3-}$, $3(C_6H_{16}N^+)$ CCDC code: UKOCEF[S31] Formula $C_{400}Au_{106}F_{240}Cl_{12}$ (UKOCEF is reported on the published paper but not in CSD)

Search 3_ The query was built using Search by Formula applying the following parameters: Au>$_{22}$ F$_{>180}$. Search on: each molecule in turn.

Output 3: 7 entries,

CCDC code: UKOCAB and UKOCEF

CCDC code: BUWJEL[S32] Formula $C_{432}H_{192}Au_{110}F_{144}^{2-}$, 2($C_6H_{16}N^+$)

CCDC code: ZIWXIP [S33] and ZIWXOV[S30] Formula $C_{180}H_{54}Au_{25}F_{108}^-$, Na^+ or $C_{24}H_{20}P^+$

CCDC code: FOYCEE[S34] and FOYCII[S34], Formula $C_{180}H_{54}Au_{24}F_{108}Pd^{2-}$ or Pt^{2-}, 2($C_{24}H_{20}P^+$), CH_2Cl_2 but the core is not only composed but gold atoms.

Search 4_ The query was built using Search by Formula applying the following parameters F$_{>180}$. Search on: sum of molecules.

Output 4: 23 entries, CCDC code: UKOCAB, AJESOA, DEMWIC, FOSPAG, GIFBIG, HIDMUF, HUZZAF, IQUFIL, JORHOQ, MOFGEW, MOFGAS, MOFGIA, MUPXAZ, OGOKUS, OQOKUC, PAHVEC, SICHUK, SICJAS, WOZWAM, WUQROQ, YUJSED, ARABON, GUGSIN. Using the "sum of molecules" the fluorinated units are mainly fluorinated anions such as $C_{16}AlF_{36}O_4^-$ or BF_4^- or $C_{32}H_{12}BF_{24}^-$ or PF_6^- those are crystallized as counter ions of large charged organic or metal-organic frameworks.

Supplementary Table 3. CSD search

CCDC code	Number of core atoms	Number of F atoms	Structure

GUGSIN	Ag_{112}	F_{306}	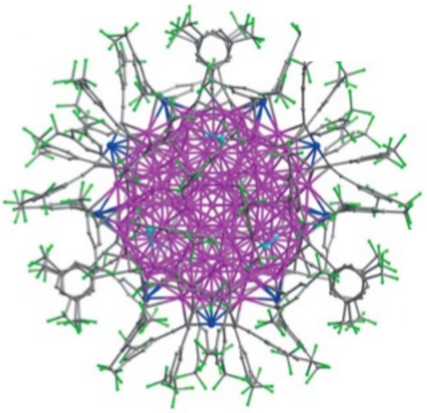
UKOCEF	Au_{106}	F_{240}	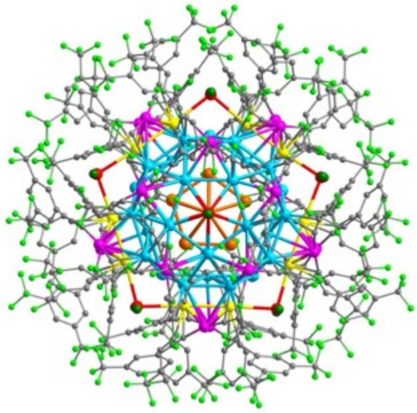
UKOCAB	Au_{67}	F_{192}	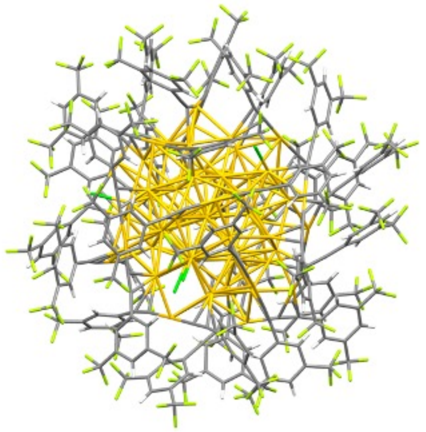
BUWJEL	Au_{110}	F_{144}	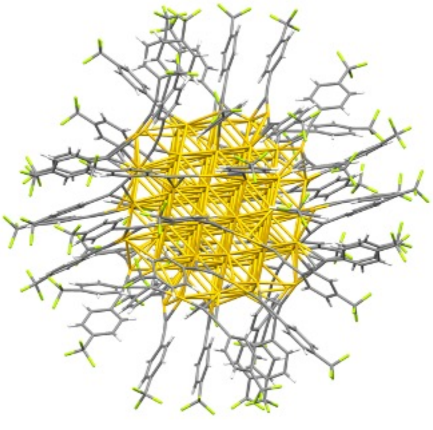

ZIWXIP	Au_{25}	F_{108}	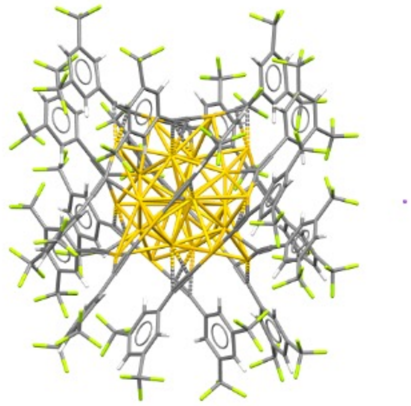
ZIWXOV	Au_{25}	F_{108}	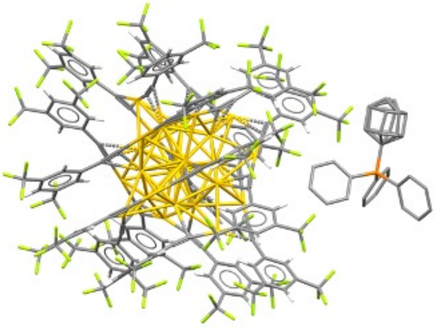
FOYCEE	$Au_{24}Pd$	F_{108}	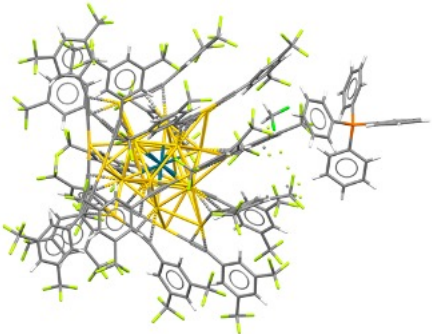
FOYCII	$Au_{24}Pt$	F_{108}	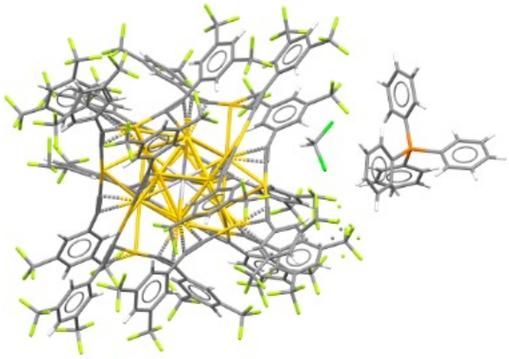

Supplementary References

[S1] X. Yue, M. B. Taraban, L. L. Hyland and Y. B. Yu, *J. Org. Chem.*, **77**, 8879-8887 (2012).

[S2] R. Mahlanen, J.-P. Jalkanen, T.A. Pakkanen, Chem. Phys. **313**, 271–277 (2005).
[S3] Y. Zhao, D.G. Truhlar, *Acc. Chem. Res.* **41**, 157–167 (2008).
[S4] T. H. Dunning Jr., *J. Chem. Phys*. **90**, 1007-23 (1989).
[S5] R. A. Kendall and T. H. Dunning Jr., *J. Chem. Phys.* **96**, 6796-806 (1992).
[S6] A. Schäfer, H. Horn, R. Ahlrichs, *J. Chem. Phys.* **97,** 2571 (1992).
[S7] S. Grimme, J. Antony, S. Ehrlich, H. Krieg, *J. Chem. Phys.* **132**, 154104 (2010).
[S8] S. F. Boys and F. Bernardi*, Mol. Phys.* **19**, 553 (1970).
[S9] M. J. Frisch, G. W. Trucks, H. B. Schlegel, G. E. Scuseria, M. A. Robb, J. R. Cheeseman, G. Scalmani, V. Barone, G. A. Petersson, H. Nakatsuji, X. Li, M. Caricato, A. Marenich, J. Bloino, B. G. Janesko, R. Gomperts, B. Mennucci, H. P. Hratchian, J. V. Ortiz, A. F. Izmaylov, J. L. Sonnenberg, D. Williams-Young, F. Ding, F. Lipparini, F. Egidi, J. Goings, B. Peng, A. Petrone, T. Henderson, D. Ranasinghe, V. G. Zakrzewski, J. Gao, N. Rega, G. Zheng, W. Liang, M. Hada, M. Ehara, K. Toyota, R. Fukuda, J. Hasegawa, M. Ishida, T. Nakajima, Y. Honda, O. Kitao, H. Nakai, T. Vreven, K. Throssell, J. A. Montgomery, Jr., J. E. Peralta, F. Ogliaro, M. Bearpark, J. J. Heyd, E. Brothers, K. N. Kudin, V. N. Staroverov, T. Keith, R. Kobayashi, J. Normand, K. Raghavachari, A. Rendell, J. C. Burant, S. S. Iyengar, J. Tomasi, M. Cossi, J. M. Millam, M. Klene, C. Adamo, R. Cammi, J. W. Ochterski, R. L. Martin, K. Morokuma, O. Farkas, J. B. Foresman, and D. J. Fox, *Gaussian 09, Revision A.02, Gaussian, Inc.*, Wallingford CT, 2016.
[S10] K. A. Wiberg, *Tetrahedron* **24**, 1083 (1968)
[S11] S. Tsuzuki, T. Uchimaru, M. Mikami, S. Urata, *J. Chem. Phys*. **116**, 3309 (2002)
[S12] R.J. Baker, P.E. Colavita, D.M. Murphy, J.A. Platts, J.D. Wallis, *J. Phys. Chem. A* **116**, 1435–1444 (2012).
[S13] C. F. Matta, N. Castillo, R.J. Boyd, *J. Phys. Chem. A* **109***,* 3669-3681(2005).
[S14] C.F. Matta and J. Hernández-Trujillo, *J. Phys. Chem. A* **107**, 7496-7504 (2003).
[S15] E.R. Johnson*, J. Am. Chem. Soc.* **132**, 18, 6498-6506 (2010).
[S16] J. Contreras-Garcia, E.R. Johnson, S. Keinan, R. Chaudret, J.-P. Piquemal, D.N. Beratan, W. Yang, *J. Chem. Theory Comput.* **7**, 625-632 (2011).
[S17] Adamo C. and Barone V., *J. Chem. Phys.* **110**, 6158-69 (1999).
[S18] Dunning Jr. T.H., *J. Chem. Phys.* **90**, 1007-23 (1989).
[S19] Kendall R. A., Dunning Jr. T. H. and Harrison R. J ., *J. Chem. Phys.* **96**, 6796-806 (1992).
[S20] A. Dass, R. Guo, J. B. Tracy, R. Balasubramanian, A. D. Douglas and R. W. Murray, *Langmuir* **24**, 310-315 (2008).

[S21] Lausi A., Polentarutti M., Onesti S., Plaisier J. R., Busetto E., Bais G., Barba L., Cassetta A., Campi G., Lamba D., Pifferi A., Mande S. C., Sarma D. D., Sharma S. M., Paolucci G., *The European Physical Journal Plus* **130**, 1-8 (2015)

[S22] Kabsch W. XDS. *Acta Crystallographica Section D* **66**, 125-132 (2010).

[S23] Sheldrick G. M. (2012). SADABS. University of Göttingen, Germany.

[S24] Sheldrick G. M., *Acta Crystallographica Section A* **71**, 3-8 (2015).

[S25] Sheldrick G. M., *Acta Crystallographica Section C* **71**, 3-8 (2015).

[S26] Emsley P., Lohkamp, B., Scott, W. G., Cowtan K., *Acta Crystallographica Section D* **66**, 486–501(2010).

[S27] Spek, A., *Acta Crystallographica Section C* **71**, 9-18 (2015).

[S28] Macrae C. F., Sovago I., Cottrell S. J., Galek P. T. A., McCabe P., Pidcock E., Platings M., Shields G. P., Stevens J. S., Towler M., Wood P. A. *J. Appl. Cryst.* **53**, 226–235 (2020).

[S29] Groom, C. R., Bruno, I. J., Lightfoot, M. P.,Ward, S. C. The Cambridge structural database. *Acta Crystallogr. Sect. B Struct. Sci. Cryst. Eng. Mater.* **72**, 171–179 (2016).

[S30] Hu, F., Li, J. J., Guan, Z. J., Yuan, S. F. & Wang, Q. M. *Angew. Chemie - Int. Ed.* **59**, 5312–5315 (2020).

[S31] Li, J. J., Guan, Z. J., Yuan, S. F., Hu, F. & Wang, Q. M. *Angew. Chemie - Int. Ed.* **60**, 6699–6703 (2021).

[S32] Wang, J. Q. *et al. J. Am. Chem. Soc.* **142**, 18086–18092 (2020).

[S33] Li, J. J., Guan, Z. J., Lei, Z., Hu, F. & Wang, Q. M. *Angew. Chemie - Int. Ed.* **58**, 1083–1087 (2019).

[S34] Takano S., Shun Ito, Tsukuda T. *J. Am. Chem. Soc.* **141**, 15994-16002 (2019).
